# Development of an Active Cable-Driven, Force-Controlled Robotic System for Walking Rehabilitation

**DOI:** 10.3389/fnbot.2021.651177

**Published:** 2021-05-21

**Authors:** Juan Fang, Michael Haldimann, Laura Marchal-Crespo, Kenneth J. Hunt

**Affiliations:** ^1^Division of Mechanical Engineering, Department of Engineering and Information Technology, Institute for Rehabilitation and Performance Technology, Bern University of Applied Sciences, Burgdorf, Switzerland; ^2^Department of Cognitive Robotics, Delft University of Technology, Delft, Netherlands; ^3^Motor Learning and Neurorehabilitation Laboratory, ARTORG Center for Biomedical Engineering Research, University of Bern, Bern, Switzerland

**Keywords:** cable-driven robots, force control, dynamic modeling, frequency-domain analysis, velocity compensation, rehabilitation robotic systems

## Abstract

In a parallel development to traditional rigid rehabilitation robotic systems, cable-driven systems are becoming popular. The robowalk expander product uses passive elastic bands in the training of the lower limbs. However, a well-controlled assistance or resistance is desirable for effective walking relearning and muscle training. To achieve well-controlled force during locomotion training with the robowalk expander, we replaced the elastic bands with actuator-driven cables and implemented force control algorithms for regulation of cable tensions. The aim of this work was to develop an active cable-driven robotic system, and to evaluate force control strategies for walking rehabilitation using frequency-domain analysis. The system parameters were determined through experiment-assisted simulation. Then force-feedback lead controllers were developed for static force tracking, and velocity-feedforward lead compensators were implemented to reduce velocity-related disturbances during walking. The technical evaluation of the active cable-driven robotic system showed that force-feedback lead controllers produced satisfactory force tracking in the static tests with a mean error of 5.5%, but in the dynamic tests, a mean error of 13.2% was observed. Further implementation of the velocity-feedforward lead compensators reduced the force tracking error to 9% in dynamic tests. With the combined control algorithms, the active cable-driven robotic system produced constant force within the four cables during walking on the treadmill, with a mean force-tracking error of 10.3%. This study demonstrates that the force control algorithms are technically feasible. The active cable-driven, force-controlled robotic system has the potential to produce user-defined assistance or resistance in rehabilitation and fitness training.

## Introduction

Diseases of or injuries to the cerebrovascular system often result in impaired sensorimotor function. Neural plasticity of the central nervous system supports that new neural connections can be developed through intensive, repetitive, and task-specific training (Maier et al., 2019). Advances in robotic technology have brought several rehabilitation robotic systems to the market. Most rehabilitation robotic systems use rigid mechanisms to assist training for the upper or lower limbs (Hesse et al., 2003; Hidler et al., 2005), including exoskeleton-based systems (e.g., Armeo Power and Lokomat from Hocoma, Switzerland) and end-effector based systems (e.g., MIT-MANUS from Interactive Motion Technologies Inc., Cambridge, Massachusetts, USA, and the G-EO system from Reha Technology, Switzerland). Along with these rigid rehabilitation robotic systems, cable-aided rehabilitation therapy has emerged in recent years with advantages including low moment of inertia, high power output, and soft human-machine contact (Rosati et al., 2017). Cable-driven rehabilitation systems available on the market include Diego (Tyromotion GmbH, Austria) for upper limb rehabilitation (Aprile et al., 2019), and the robowalk expander system (h/p/cosmos Sports & Medical GmbH, Germany) for lower limb training (Schulze et al., 2019).

The robowalk expander product is a system using passive elastic bands in the training of the lower limbs. It includes two supporting frames which can be fixed on a treadmill, and four rubber bands which can be attached to the thigh, shank, or ankle segments via cuffs. Depending on the band setup and pre-tension adjustment, the robowalk expander system can provide assistance or resistance to the legs during walking/running on a treadmill (Schulze et al., 2019). For patients who have walking impairments, assistance from the elastic bands can help them to walk on the treadmill with improved speed and stride length (Beckmann-Hemmers, 2012; Jöllenbeck and Pietschmann, 2018). However, the passive assistance provided by the unactuated elastic bands is dependent on the leg position. Due to the factors of different temperatures and varying rubber elasticity after long-term usage (Beckmann-Hemmers, 2012), the force provided by the elastic band might change non-linearly with the leg position. A well-controlled assistance or resistance, which allows patients to learn how to exert force to walk correctly and thereby to bring improved walking skills (Wu et al., 2011), is believed to be desirable for effective walking relearning and muscle training.

In order to provide well-controlled guidance, several cable-driven rehabilitation robotic systems were developed, with various control algorithms investigated. The cable-driven locomotor trainer (CaLT) used four cable-driven units to control the end-effector ankle segments, where two units were connected jointly on one ankle segment. Using a proportional-derivative (PD) controller for position control, CaLT produced assistance and resistance during walking without changing the ankle trajectory (Wu et al., 2011). Tests on patients with incomplete spinal cord injury showed that increased overground walking speed was obtained after 8 weeks of training using CaLT. Agrawal et al. designed a cable-driven active leg exoskeleton (C-ALEX) for walking rehabilitation (Jin et al., 2015). Cable-driven active leg exoskeleton included two cable-driven units for the right thigh and another two cable-driven units for the right shank segment. Using proportional–integral–derivative (PID) algorithms, friction compensation and a feedforward element, C-ALEX produced a well-controlled ankle trajectory in the right leg during walking. The Robotic Physical Exercise and System (ROPES) included two spring-assisted cable-driven units (one connected to the left thigh, and the other to the left shank) and two cable-driven units (both connected to the left foot) (Alamdari and Krovi, 2015a). Using PD algorithms, forward kinematics, and gravity compensation for position control, ROPES produced walking-like movement in the left hip, knee, and ankle joints.

Apart from trajectory tracking, several studies investigated control strategies for force regulation in cable-driven robotic systems, where motion-induced disturbances require additional compensation. Zou et al. developed a lower limb rehabilitation system with eight cable-driven units, where two units were used to control each thigh and each shank of both legs (Zou et al., 2019). Frequency-domain analysis revealed that the system structure including the moment of inertia and cable stiffness influenced the dynamics of the cable-driven robotic system significantly (Zou et al., 2019). The force control strategy comprised an integral term, a lead compensator and a feedforward correction element. Experimental results from static tests showed that the system produced forces that tracked sinusoidal force references with different frequencies. In contrast to force control in a static situation, force control during movement had further disturbance from the actuator velocity (Zou et al., 2018a, 2019). To compensate such velocity-related disturbances, a force compensator using the structure invariance principle was investigated (Zhang et al., 2017; Zou et al., 2018a,b; Wang et al., 2020), while disturbance observers for force control were also implemented (Jung and Bae, 2016; Wang et al., 2019). These force control algorithms, combined with disturbance compensation, often result in complex control strategies.

To achieve well-controlled force during locomotion training with the robowalk expander, we replaced the elastic bands with actuator-driven cables and implemented novel force control algorithms for regulation of cable tensions. It was observed here that movement-induced disturbances influenced the force-tracking performance significantly. Velocity disturbance is a common issue in force-controlled cable-driven robotic systems, but how to compensate the velocity disturbance is the key issue. The disturbance compensators described in the literature (Jung and Bae, 2016; Zhang et al., 2017; Zou et al., 2018a,b; Wang et al., 2019, 2020), due to different system dynamics, could not be easily applied in the current study. The current work presented a novel approach to address this issue. By analyzing the kinetic model of the cable-driven system and estimating the system parameters, this paper introduced lead control algorithms for force control and also for velocity compensation. The methods of how to determine the suitable parameters for the lead control algorithms were deduced in detail using frequency-domain analysis. A comprehensive search in the databases of PubMed, Web of Science and Google Scholar using search items of “force control AND cable-driven,” “lead controller OR lead compensator,” “velocity compensation OR speed compensation” failed to yield any similar control strategies developed in the current work. The lead control algorithms were straightforwardly developed and produced satisfactory force tracking with negligible velocity-related disturbances during walking. After being validated in the current study, this novel control strategy has potential to be generally applied in force-controlled cable-driven robotic systems.

The aim of this work was to develop an active cable-driven robotic system, and to evaluate force control strategies for walking rehabilitation using frequency-domain analysis. The active cable-driven, force-controlled robotic system has the potential to produce user-defined assistance or resistance in rehabilitation and fitness training.

## Methods

### The Active Cable-Driven Robotic System

**Figure 1:**
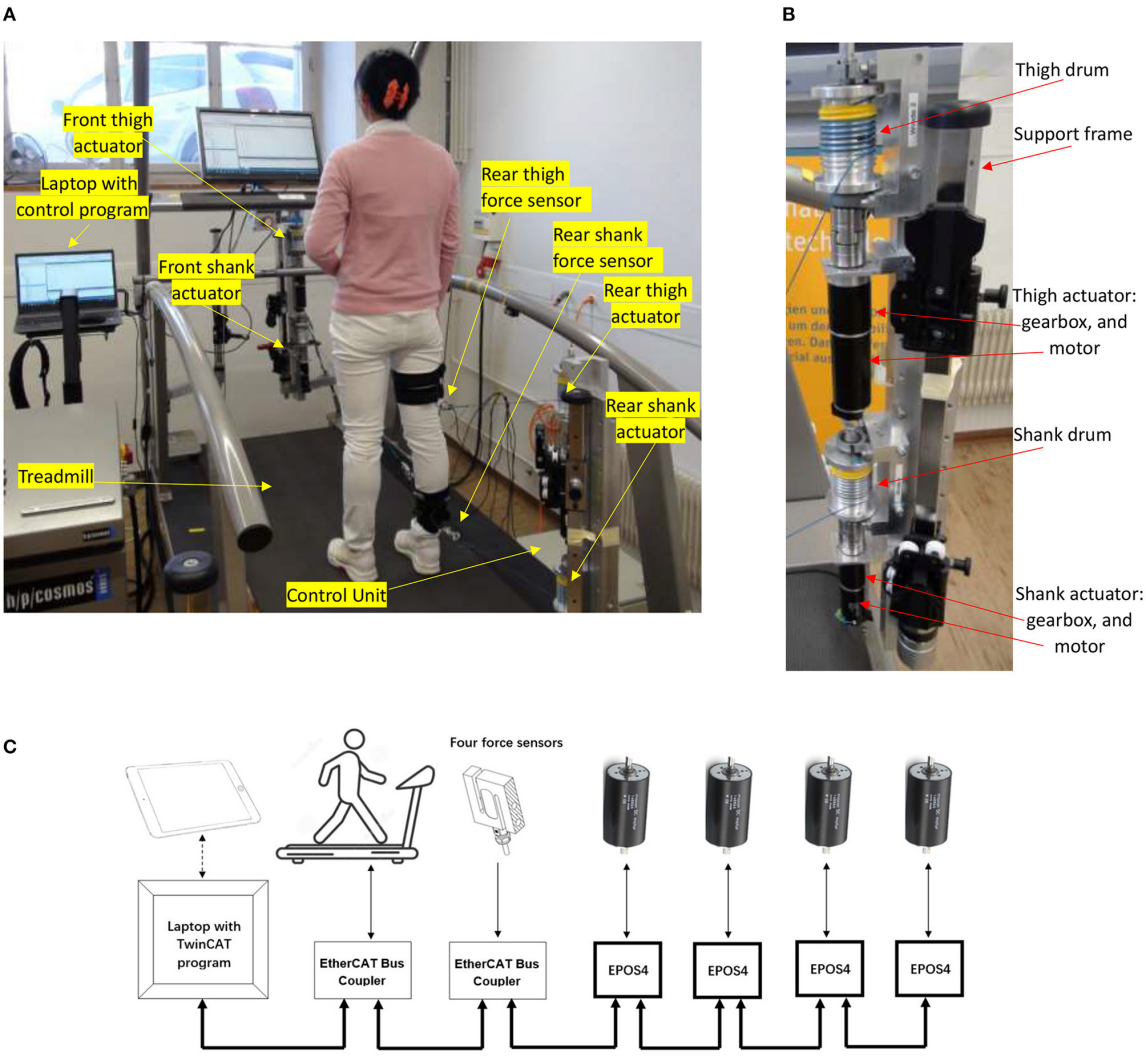
The active cable-driven robotic system. **(A)** The physical system with a participant. The modified robowalk expander units are at the front-right and rear-right. **(B)** Modified expander unit with support frame and two actuators (rear-right). **(C)** The communication structure.

Two robowalk expander units were fixed on a treadmill (model Venus, h/p/cosmos Sports & Medical GmbH, Germany), with one at the front and one at the rear (Figure 1A). The treadmill speed was controlled by a computer via an RS-232 serial port. In order to achieve well-controlled active forces, four rigid cables were used and four motor-drum actuators were designed (Figure 1B) to control the cable tensions. Two cable-driven units were mounted on the front-right support frame of the expander unit, while the other two were mounted on the rear-right support frame (Figures 1A,B).

Two actuator assemblies (DC motor model RE50, gearhead with a ratio of 91, Encoder HEDL 5540; maxon motor, Switzerland) pulled the right thigh while moving forwards and backwards, and two actuator assemblies (DC motor model RE40, gearhead with a ratio of 113, Encoder HEDL 5540; maxon motor, Switzerland) pulled the right shank similarly. Two servocontrollers (EPOS4 70/15; maxon motor, Switzerland) sent torque commands to the thigh actuators, and two further servocontrollers (EPOS4 50/5; maxon motor, Switzerland) controlled the shank actuators. A shunt regulator (DSR 70/30; maxon motor, Switzerland) was used to buffer energy generated when the motors worked as generators. For safety, an emergency stop was fixed on the right horizontal handrail (see Supplementary Material “Walk with the active cable-driven robotic system. mp4”). The servocontrollers provide a Safe Torque Off (STO) function, which brings the actuator to a torque-free, safe condition when the stop command is received. To enhance safety, the wires for the inputs of the STO function were connected to the emergency stop of the treadmill. By this means, activating the emergency stop button stops the treadmill immediately, and simultaneously sets the four actuators into the zero-torque-output safe mode.

Force sensors with range −500 to 500 N were connected in series in each cable close to the leg fixation points (Figure 1A). The two force sensors for the thigh segment (Bengbu Sensor Company, China; resolution 0.00012 N) were recorded using an analog input card (ELM3144-0000, Beckhoff Automation GmbH & Co, Germany). The two force sensors for the shank segment (KD40S, Transmetra GmbH, Switzerland; resolution 0.00003 N) were recorded using an amplifier/converter interface (EL3356-0010, Beckhoff Automation GmbH & Co, Germany).

The real-time control program was developed using TwinCAT3 software (Beckhoff Automation GmbH & Co., Germany) running in a laptop at a sample rate of 1,000 Hz (Figure 1C). The control program sent speed commands to and received the speed information from the treadmill via a serial interface (KL6001, Beckhoff Automation GmbH & Co., Germany), which was installed on the EtherCAT Bus Coupler (BK1120, Beckhoff Automation GmbH & Co., Germany). The force information was transferred to the control program via an EtherCAT coupler (EK1100, Beckhoff Automation GmbH & Co., Germany). Four EtherCAT cards (Part No. 581245, maxon motor, Switzerland) were used to establish the communication between the servocontrollers and the TwinCAT program (Figure 1C). TC3 Controller Toolbox in TwinCAT3 was used to develop the control algorithms. A live-view Human Machine Interface (HMI) was developed using TC3 HMI Engineering toolbox, which allowed operation of the active cable-driven robotic system via a tablet. TC3 Measurement Toolbox was used to present, analyze, save, and export the experimental data, including the reference and actual force, actuator control signals, and velocities.

### Control Development

#### Dynamics of the Active Cable-Driven Robotic System

**Figure 2:**
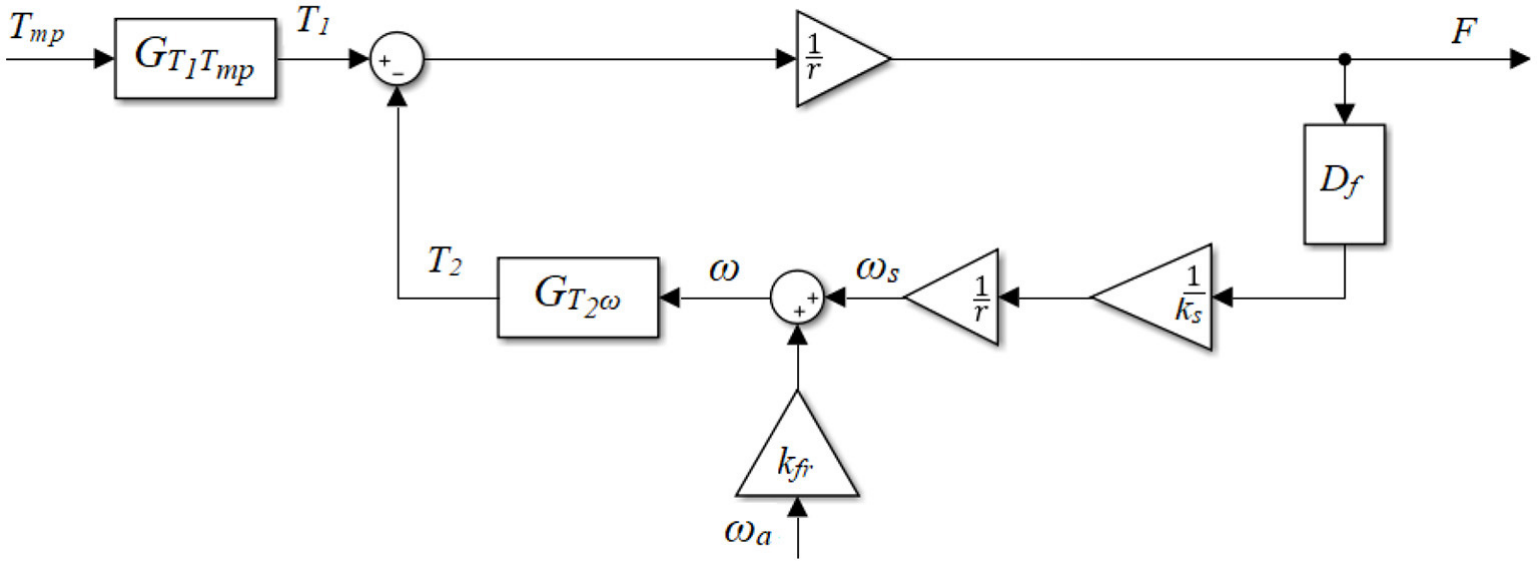
Block diagram of the active cable-driven robotic system.

A model of the active cable-driven robotic system was developed in Matlab/Simulink (The Mathworks, Inc., Natick, USA). The cable was considered as a spring with a stiffness of *k*_*s*_, while the weight and damping of the cable were neglected. The dynamic model of one cable-actuator unit in the active cable-driven robotic system is depicted (Figure 2).

*T*_1_ is the total torque that the actuator (the motor and the gearbox) generates. The servocontroller sent the torque command as a percentage of the maximal torque *T*_*mp*_. The transfer function of the actuator linking the torque command *T*_*mp*_ to the output torque *T*_1_, was expressed as a linear first-order transfer function:

(1) $$\begin{array}{l} T_{mp}\to T_{1}:G_{T_{1}T_{mp}}\left(s\right)=\frac{k_{a}}{\tau_{a}s+1}, \end{array}$$

where τ_*a*_ is the time constant and was determined by the setting of the servocontrollers, while *k*_*a*_ is the steady-state gain and was determined using the motor and gearbox specifications. *T*_2_ is the torque used to compensate the viscous component of the actuator, which is a function of the final angular velocity of the actuator ω:

(2) $$\begin{array}{l} \omega \to T_{2}:G_{T_{2}\omega }=J_{a}D_{f}+B_{a}, \end{array}$$

where *J*_*a*_ and *B*_*a*_ are the moment of inertia and damping of the actuator. *D*_*f*_ is the derivative term in combination with a low pass filter, which is:

(3) $$\begin{array}{l} D_{f}\left(s\right)=\frac{s}{\frac{1}{\omega_{lp}}s+1}. \end{array}$$

The cut-off frequency ω_*lp*_ was set to be 5 × 10^4^ rad/s (*f*_*lp*_ ≈ 8,000 Hz), which is much higher than the system sampling frequency of 1,000 Hz, therefore the filter will not influence simulation of the system dynamics within the frequencies that are of interest in this study.

The final angular velocity of the actuator ω is primarily influenced by two variables: the force-related velocity ω_*s*_ and the motion-produced velocity ω_*a*_. The force *F* on the spring with a stiffness of *k*_*s*_ results in an angular velocity of the actuator, ω_*s*_:

(4) $$\begin{array}{l} F\to \omega_{s}:G_{\omega_{s}F}=\frac{D_{f}}{rk_{s}}, \end{array}$$

where *D*_*f*_ is the same derivative term in combination with a low pass filter, as described in Equation (3). The leg motion, i.e., the walking movement, is related to the angular velocity of the actuator. This velocity was approximated by the measured actuator velocity ω_*a*_, which is the motor velocity divided by the gear ratio.

Due to the contact stress between the cable and the drum, the system has complex friction dynamics. The dynamic friction in general is related to the leg-motion induced velocity ω_*a*_. Thus, a velocity scale *k*_*fr*_ was introduced (Figure 2). This scaled velocity passes through the actuator dynamics, i.e., Equation (2), representing the friction between the cable and the drum, apart from the leg-motion required torque. Thus, the final angular velocity ω is

(5) $$\begin{array}{l} \omega =\omega_{s}+k_{fr}\omega_{a}. \end{array}$$

The force output *F* of the active cable-driven robotic system, i.e., the cable tension is

(6) $$\begin{array}{l} F=\frac{T_{1}-T_{2}}{r}, \end{array}$$

where *r* is the drum radius.

The active cable-driven robotic system has two inputs driving the final force output, which are the torque command of the actuator *T*_*mp*_ and the actuator velocity ω_*a*_. This study considered the torque command *T*_*mp*_ as the main input, while the actuator velocity ω_*a*_ was regarded as a plant disturbance. The transfer function from the actuator control signal to the force output, which is regarded as the plant open-loop transfer function, is:

(7) $$\begin{array}{l} T_{mp}\to F:G_{FT_{mp}}=\frac{G_{T_{1}T_{mp}}}{r+\frac{D_{f}G_{T_{2}\omega }}{k_{s}r}}. \end{array}$$

The transfer function from the disturbance, i.e., the angular velocity of the actuator, to the force output is:

(8) $$\begin{array}{l} \omega_{a}\to F:G_{F\omega_{a}}^{A}=-\frac{k_{fr}G_{T_{2}\omega }}{r+\frac{D_{f}G_{T_{2}\omega }}{k_{s}r}}. \end{array}$$

#### System Parameter Determination

The parameters τ_*a*_ and *k*_*a*_ (Equation 1) were determined using the hardware controller settings, while *J*_*a*_ (Equation 2) and *r* (Equations 4, 6) were determined from the geometric configuration. The remaining parameters *B*_*a*_ (Equation 2), *k*_*s*_ (Equation 4), and *k*_*fr*_ (Equation 5) were determined using the experiment-assisted simulation approach described below.

**Figure 3:**
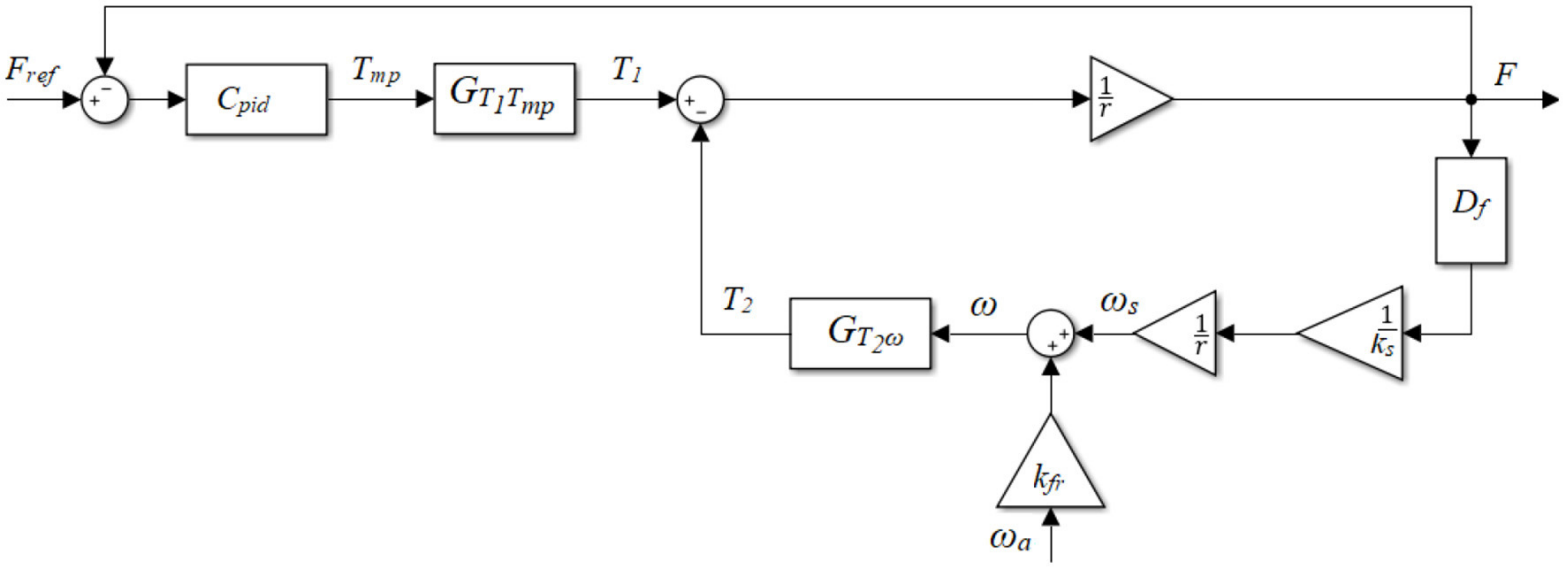
Block diagram of the plant with the tentative PID controller.

In order to determine *k*_*fr*_, the system has to move at different speeds. However, due to the high friction, it is difficult to pull the cable in the open-loop active cable-driven robotic system to make the actuators move. A tentative closed-loop force controller (Figure 3) was therefore developed for each actuator. Conventional closed-loop controllers such as PD algorithms were reported in studies (Marchal-Crespo et al., 2012; Alamdari and Krovi, 2015b). A pretest using a PD controller showed steady-state errors, therefore a PID controller was implemented for each actuator as the tentative controller *C*_*pid*_ in this phase (Figure 3):

(9) $$\begin{array}{l} C_{pid}\left(s\right)=k_{p}+\frac{k_{i}}{s}+k_{d}\frac{s}{\tau_{d}s+1}, \end{array}$$

where *k*_*p*_, *k*_*i*_, and *k*_*d*_ are the gains for the proportional, integral, and derivative terms. The derivative term was implemented with a low pass filter, where τ_*d*_ is the time constant of the low pass filter.

In the parameter determination phase, static and dynamic tests were implemented on a female participant with a height of 1.6 m and a mass of 53 kg. During the test setup, she stood on the treadmill with her right thigh and shank segments attached to the actuator-driven cables via cuffs, while her left leg was free (Figure 1A). Each cable was connected to an independent cuff to prevent interaction between the front and rear actuators. Square-wave and sinusoidal force references were implemented. By resisting the force from the cables throughout the test, the participant tried to stand still on the static treadmill in the static test, while in the dynamic test she tried to walk normally on the treadmill which ran at different speeds.

A Matlab/Simulink model simulating four actuators with the tentative PID controllers of the active cable-driven robotic system was developed (Figure 3). The experiment-used reference *F*_*ref*_ and the experiment-recorded actuator velocity ω_*a*_ were used as the inputs in the model. Using the calculated parameters τ_*a*_, *k*_*a*_, *J*_*a*_, and *r*, and trying different possible values of *B*_*a*_, *k*_*s*_, and *k*_*fr*_, the model simulated the dynamics of the PID-controlled active cable-driven robotic system. The simulated force output *F*_sim_ was then compared with the experimental force *F*_exp._ It should be noted that the current simulation task was to determine the suitable *B*_*a*_, *k*_*s*_, and *k*_*fr*_, which yielded the simulated force *F*_sim_ as close as possible to the experimental data *F*_exp_. As the active cable-driven robotic system focused on walking training, the goodness of fit of the force output during the dynamic test was specifically investigated using a normalized root-mean-square error (NRMSE) on an evaluation interval from *t*_*o*_ to *t*_1_:

(10) $$\begin{array}{l} \text{NRMSE}=(1-\sqrt{\frac{\overset{t_{1}}{\underset{t=t_{0}}{\sum }}{\left(F_{\exp}\left(t\right)-F_{sim}\left(t\right)\right)}^{2}}{\overset{t_{1}}{\underset{t=t_{0}}{\sum }}{\left(F_{\exp}\left(t\right)-\bar{F_{\exp}\left(t\right)}\right)}^{2}}})\times 100\%, \end{array}$$

where $\bar{F_{\exp}}$ is the mean of *F*_exp_ between *t*_*o*_ and *t*_1_. The values of *B*_*a*_, *k*_*s*_, and *k*_*fr*_ that yielded an NRMSE higher than 60% were considered suitable parameters for the active cable-driven robotic system.

#### Force Control Strategy

**Figure 4:**
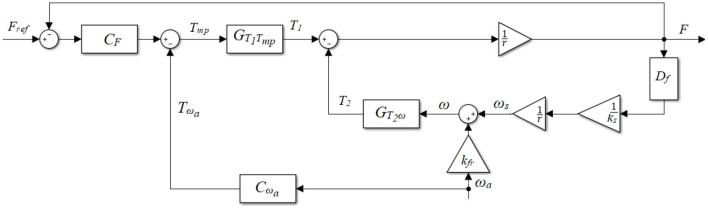
Block diagram of the plant with the force-feedback lead controller *C*_*F*_ and the velocity-feedforward lead compensator *C*_ω_*a*__.

The plant model was used to develop accurate force control strategies. The force control structure (Figure 4) included a force-feedback lead controller and a velocity-feedforward lead compensator for each actuator.

##### Force-Feedback Lead Controller

**Figure 5:**
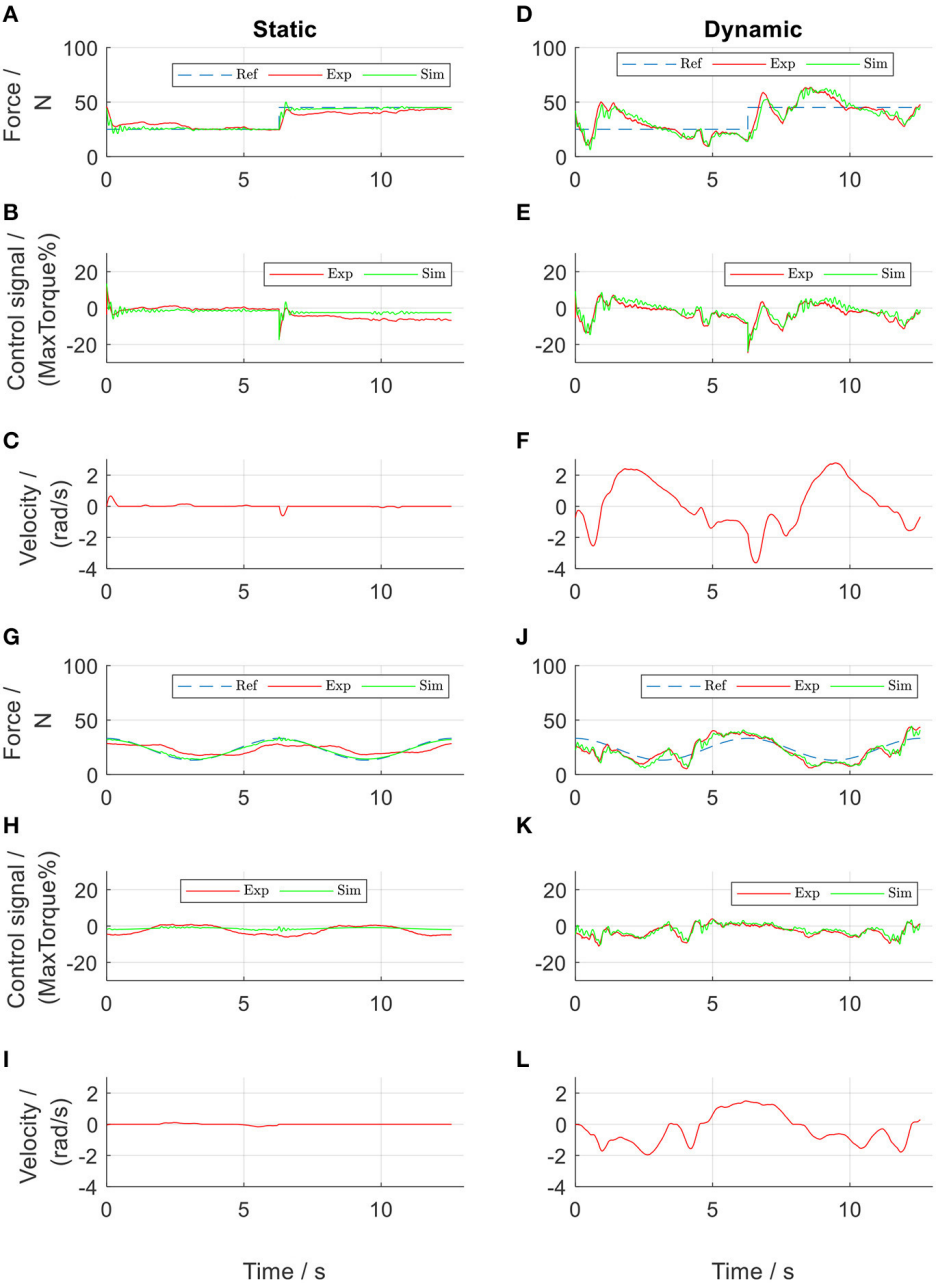
PID-regulated force control of the front thigh actuator. Subplots **(A–F)** show results from square-wave reference test, while subplots **(G–L)** show results from sinusoidal reference test. Ref, references; Exp, experimental values; Sim, simulation results.

As the tests in the parameter determination phase showed an obvious delay (e.g., Figure 5G), a lead compensator *C*_*F*_ was developed initially (Figure 4, with *C*_ω_*a*__ = 0):

(11) $$\begin{array}{l} C_{F}\left(s\right)=\;k_{F}\frac{\tau_{F}s+1}{a_{F}\tau_{F}s+1}, \end{array}$$

where *k*_*F*_ is the gain, and *a*_*F*_ < 1. The controller parameters *a*_*F*_ and τ_*F*_ are linked with the center frequency *f*_*F*_ by

(12) $$\begin{array}{l} \tau_{F}=\frac{1}{f_{F}\sqrt{a_{F}}}. \end{array}$$

The center frequency *f*_*F*_ is the frequency when the lead compensator *C*_*F*_ has the largest phase. The transfer function from the reference *F*_*ref*_ to the force output *F* is therefore:

(13) $$\begin{array}{l} F_{ref}\to F:G_{FF_{ref}}=\frac{C_{F}G_{FT_{mp}}}{1+C_{F}G_{FT_{mp}}}. \end{array}$$

Furthermore, the transfer function from *F*_*ref*_ to the control signal *T*_*mp*_ is:

(14) $$\begin{array}{l} F_{ref}\to T_{mp}:G_{T_{mp}F_{ref}}=\frac{C_{F}}{1+C_{F}G_{FT_{mp}}}. \end{array}$$

The transfer function from the disturbance term (angular velocity ω_*a*_) to the force output *F* is:

(15) $$\begin{array}{l} \omega_{a}\to F:G_{F\omega_{a}}^{B}=\frac{G_{F\omega_{a}}^{A}}{1+C_{F}G_{FT_{mp}}}=-\frac{k_{fr}G_{T_{2}\omega }}{G_{T_{1}T_{mp}}C_{F}+r+\frac{D_{f}G_{T_{2}\omega }}{k_{s}r}}. \end{array}$$

To evaluate the stability of the feedback system, Nyquist analysis was performed using the loop gain *G*_*Lo*_:

(16) $$\begin{array}{l} G_{L_{o}}=C_{F}G_{FT_{mp}}. \end{array}$$

Apart from the gain and phase margins, the modulus margin was calculated, which is the minimal distance between the critical point [−1, 0] and the Nyquist plot *G*_*Lo*_ (Aström and Murray, 2010).

##### Velocity-Feedforward Lead Compensator

The velocity compensator introduces an extra input *T*_ω_*a*__ to the torque command (Figure 4), with the aim of reducing the disturbance on the force output induced by the actuator velocity. In this case, the influence of *T*_ω_*a*__ on the force output is:

(17) $$\begin{array}{l} T_{\omega_{a}}\to F:G_{FT_{\omega_{a}}}=\frac{G_{FT_{mp}}}{1+C_{F}G_{FT_{mp}}}=-\frac{G_{T_{1}T_{mp}}}{C_{F}G_{T_{1}T_{mp}}+r+\frac{D_{f}G_{T_{2}\omega }}{k_{s}r}}. \end{array}$$

Experimental tests in section System Parameter Determination showed that the actual force output followed a similar shape of the angular velocity curve, but with some phase shifts (comparing Figures 5D,F, as well as Figures 5J,L). Therefore, the angular velocity was used as the input for the compensator channel, and a feedforward lead compensator *C*_ω_*a*__ was further implemented (Figure 4):

(18) $$\begin{array}{l} C_{\omega_{a}}\left(s\right)=k_{\omega_{a}}\frac{\tau_{\omega_{a}}s+1}{a_{\omega_{a}}\tau_{\omega_{a}}s+1}, \end{array}$$

where *k*_ω_*a*__ is the gain, and *a*_ω_*a*__ < 1. The control parameters *a*_ω_*a*__, τ_ω_*a*__ and the center frequency *f*_ω_*a*__ are interlinked:

(19) $$\begin{array}{l} \tau_{\omega_{a}}=\frac{1}{f_{\omega_{a}}\sqrt{a_{\omega_{a}}}}. \end{array}$$

With this compensator *C*_ω_*a*__, the transfer function of the velocity compensation channel is

(20) $$\begin{array}{l} \omega_{a}\to F:G_{F\omega_{a}}^{C}=G_{FT_{\omega_{a}}}C_{\omega_{a}}, \end{array}$$

where *G*_*FT*_ω_*a*___ is given by Equation (17).

With the force-feedback controller and the velocity-feedforward compensator, the overall transfer functions from the angular velocity ω_*a*_ to the force output *F* and to the torque command *T*_*mp*_, are, respectively:

(21) $$\begin{array}{l} \omega_{a}\to F:G_{F\omega_{a}}^{D}=\frac{G_{F\omega_{a}}^{A}+C_{\omega_{a}}G_{FT_{mp}}}{1+C_{F}G_{FT_{mp}}}\\ \;=-\frac{G_{T_{1}T_{mp}}C_{\omega_{a}}+k_{fr}G_{T_{2}\omega }}{C_{F}G_{T_{1}T_{mp}}+r+\frac{D_{f}G_{T_{2}\omega }}{k_{s}r}}. \end{array}$$

(22) $$\begin{array}{l} \omega_{a}\to T_{mp}:G_{T_{mp}\omega_{a}}=-\frac{C_{F}G_{F\omega_{a}}^{A}+C_{\omega_{a}}}{1+C_{F}G_{FT_{mp}}}\\ \;=\frac{k_{fr}G_{T_{2}\omega }-\frac{C_{\omega_{a}}\left(D_{f}G_{T_{2}\omega }+k_{s}r^{2}\right)}{k_{s}rC_{F}}}{G_{T_{1}T_{mp}}+\frac{D_{f}G_{T_{2}\omega }+k_{s}r^{2}}{k_{s}rC_{F}}}. \end{array}$$

#### Simulation and Evaluation of the Control Algorithms

A model was developed in Matlab/Simulink to design, simulate, and evaluate the force control algorithms for the four actuators (developed in section Force Control Strategy). Using the system parameters determined in section System Parameter Determination, two dynamic cases of the active cable-driven robotic system were simulated: (i) the nominal plant without consideration of the velocity disturbance; and (ii) the plant with consideration of the velocity disturbance. In simulation (i), only the closed-loop force-feedback controller *C*_*F*_ was simulated (Figure 4) with *C*_ω_*a*__ = 0 and ω_a_ = 0. The simulated force output *F*_nom_ served as the target for the force control. Simulation (ii) used the force-feedback lead controller *C*_*F*_ and the velocity-feedforward lead compensator *C*_ω_*a*__ (Figure 4), where the actuator velocity when the participant walked at 0.2 m/s was used as the velocity input ω_*a*_. Different control parameters, i.e., *k*_*F*_, *a*_*F*_, and τ_*F*_ for the force-feedback controller *C*_*F*_, and *k*_ω_*a*__, *a*_ω_*a*__, and τ_ω_*a*__ for the velocity-feedforward compensator *C*_ω_*a*__, were simulated, with the aim of producing force outputs that well-tracked square-wave and sinusoidal reference forces. To evaluate the force tracking accuracy, the root-mean-square error (RMSE) between the simulated force output *F*_sim_ from simulation (ii) and the nominal force output *F*_nom_ from simulation (i) was calculated on an evaluation interval from *t*_*o*_ to *t*_1_ as follows:

(23) $$\begin{array}{l} \text{RMS}{\text{E}}_{F\text{sim}}=\sqrt{\frac{1}{N}\overset{t_{1}}{\underset{t=t_{0}}{\sum }}{\left(F_{\text{ sim}}\left(t\right)-F_{\text{ nom}}\left(t\right)\right)}^{2}}, \end{array}$$

where *N* is the number of data points in the interval *t*_*o*_ and *t*_1_. When the simulated force output had an RMSE_F*sim*_ < 5 N, which was considered satisfactory, the control algorithms were implemented in the four actuators of the active cable-driven robotic system for evaluation.

The evaluation tests were performed on the same participant who performed the tests in section System Parameter Determination and included two sections: “static” tests where the participant stood still, and “dynamic” tests where the participant walked at 0.2 m/s. Both tests used the square-wave and sinusoidal force references and implemented two control algorithms: (1) using the force-feedback controllers *C*_*F*_ only (Figure 4, with *C*_ω_*a*__ = 0), and (2) using the force-feedback controllers *C*_*F*_ and the velocity-feedforward compensators *C*_ω_*a*__ (Figure 4). Replacing *F*_sim_ with *F*_exp_ in Equation (23) yielded the difference between the experimental and nominal force, RMSE_F*exp*_. To enable comparison between different force strategies, the experimental force-tracking error is presented in percentage terms after RMSE_F*exp*_ was divided by the maximal reference force.

After the force control algorithms were evaluated to be feasible in four actuators, the participant finally walked at 0.2 m/s with a constant reference force within the four cables in the active cable-driven robotic system.

## Results

**Figure 6:**
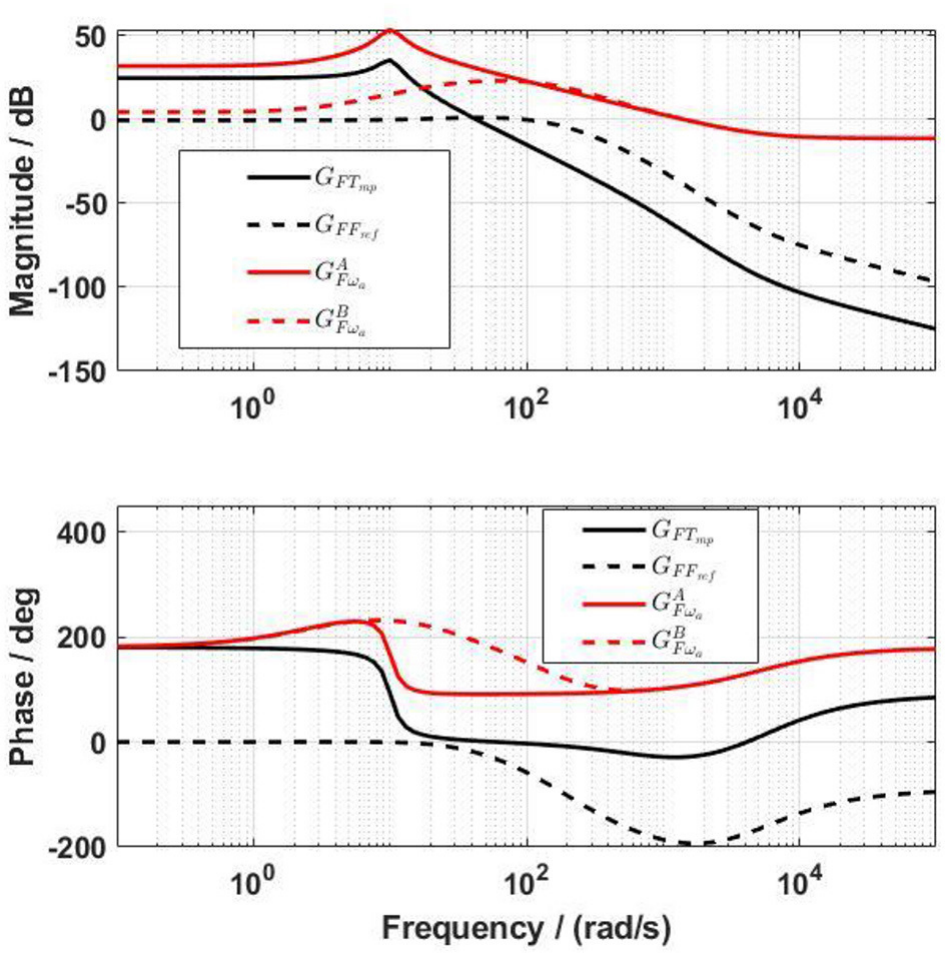
Bode diagram of the transfer functions from the actuator control signal to the force output before (*G*_*FT*_*mp*__) and after (*G*_*FF*_*ref*__) including the force-feedback lead controller, and also the transfer functions from the angular velocity of the actuator to the force output before ($G_{F\omega_{a}}^{A}$) and after ($G_{F\omega_{a}}^{B}$) including the force-feedback lead controller.

**Figure 7:**
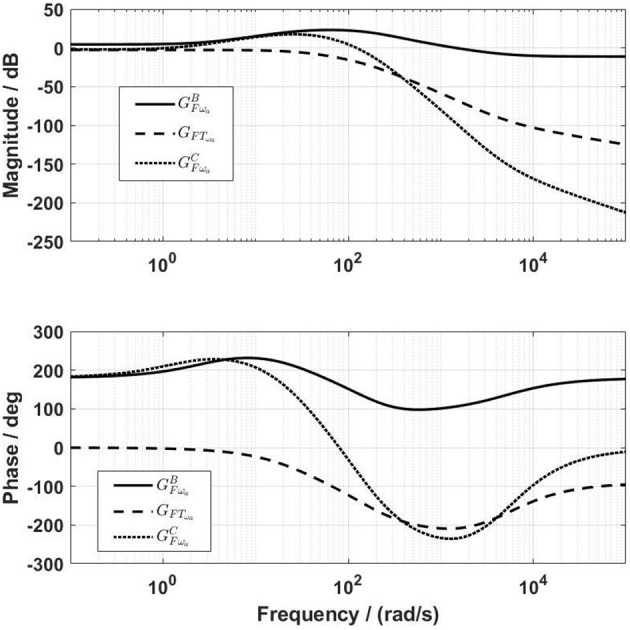
Frequency characteristics of the compensation channel before (*G*_*FT*_ω_*a*___) and after ($G_{F\omega_{a}}^{C}$) the inclusion of the velocity-feedforward lead compensator. The amplitude of $G_{F\omega_{a}}^{C}$ is close to $G_{F\omega_{a}}^{B}$ for frequencies up to 40 rad/s.

**Figure 8:**
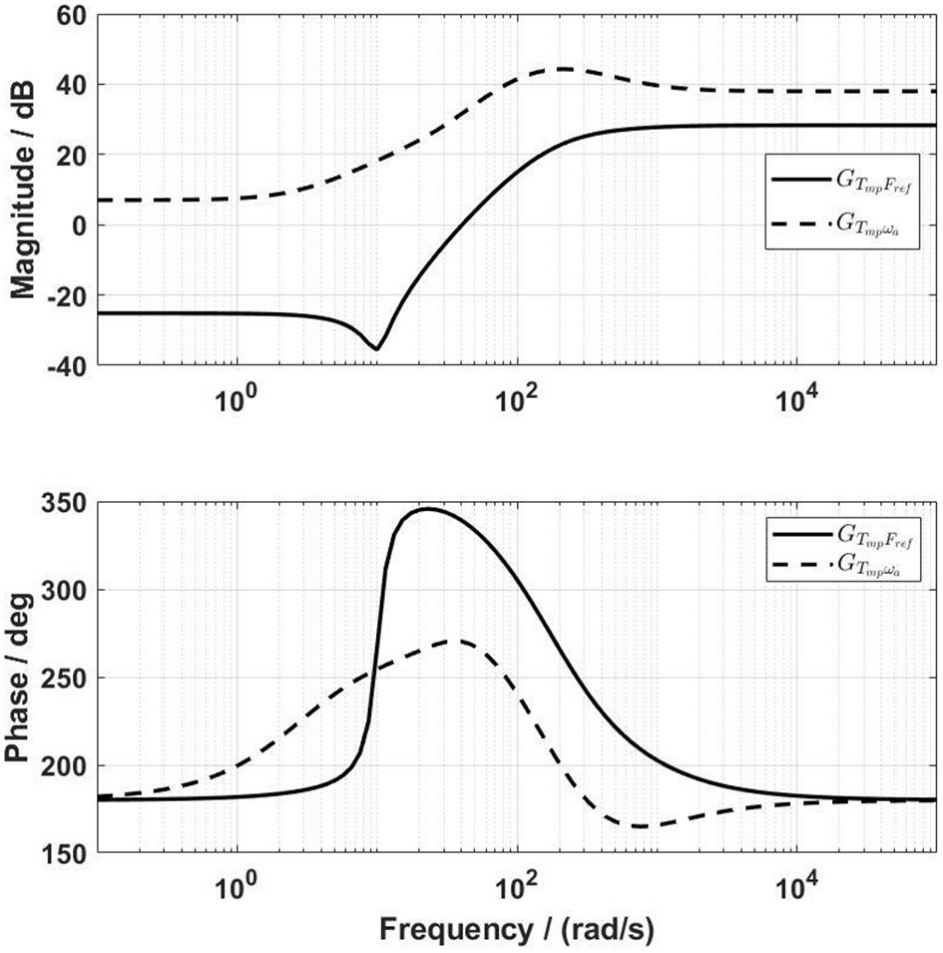
Frequency characteristics of the transfer functions from the reference *F*_*ref*_ and from the angular velocity ω_*a*_ to the control signal *T*_*mp*_.

**Figure 9:**
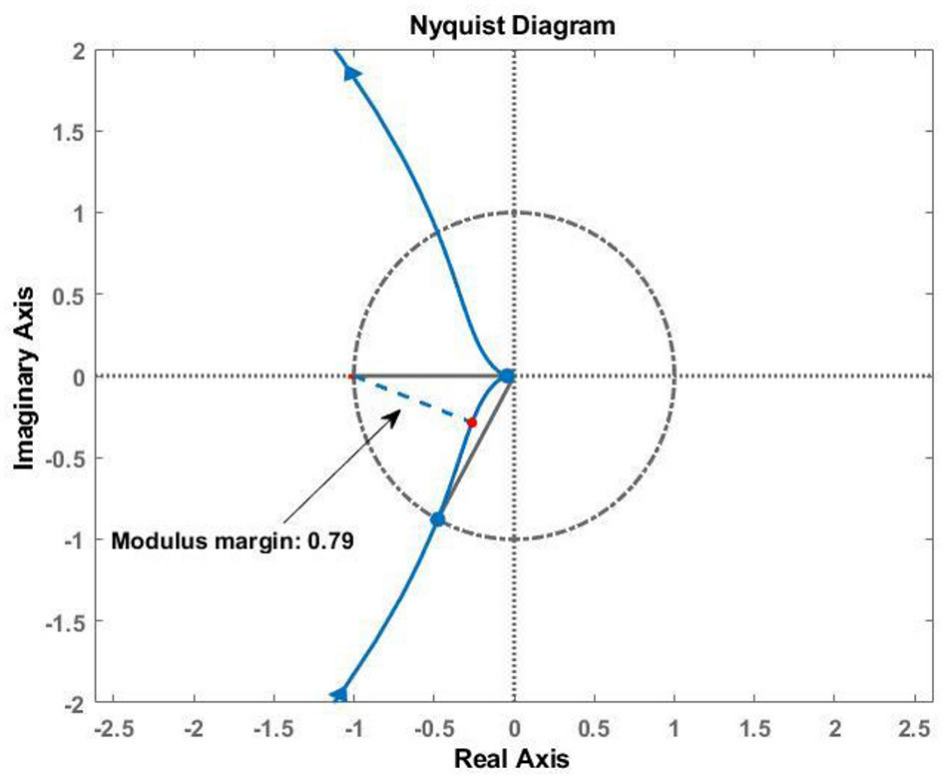
Stability analysis of the closed-loop system using loop gain *G*_*Lo*_ (Equation 16). The gain margin is 27.4 dB (at 824 rad/s), and the phase margin is 61.5° (at 103 rad/s). The blue dashed line shows the modulus margin of 0.79.

**Figure 10:**
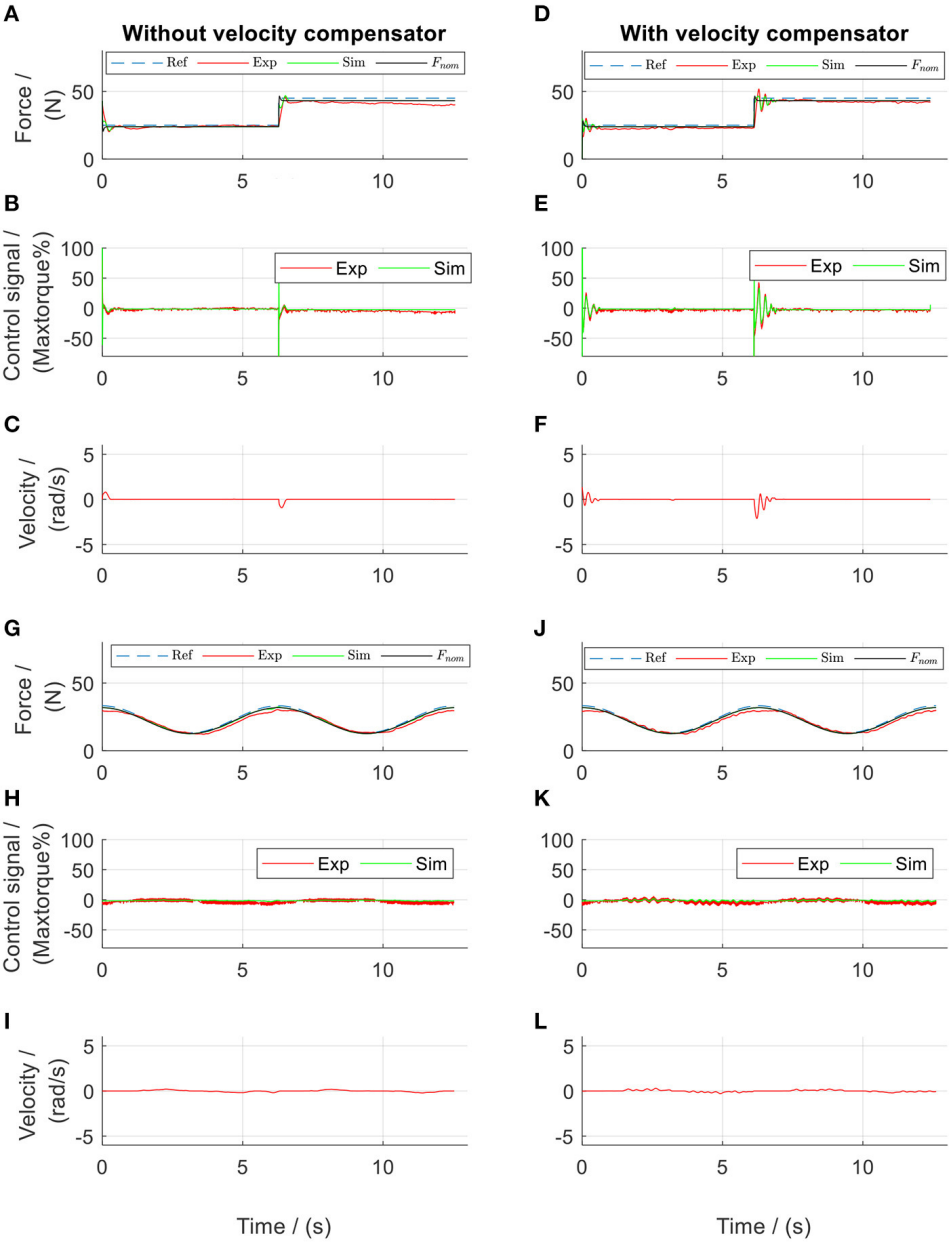
Lead algorithms regulated force control of the front thigh actuator in the static tests. Subplots **(A–F)** show the results from square-wave reference test, while subplots **(G–L)** show the results from sinusoidal reference test. Ref, references; Exp, experimental values; Sim, simulation results; *F*_nom_, the simulated target.

**Figure 11:**
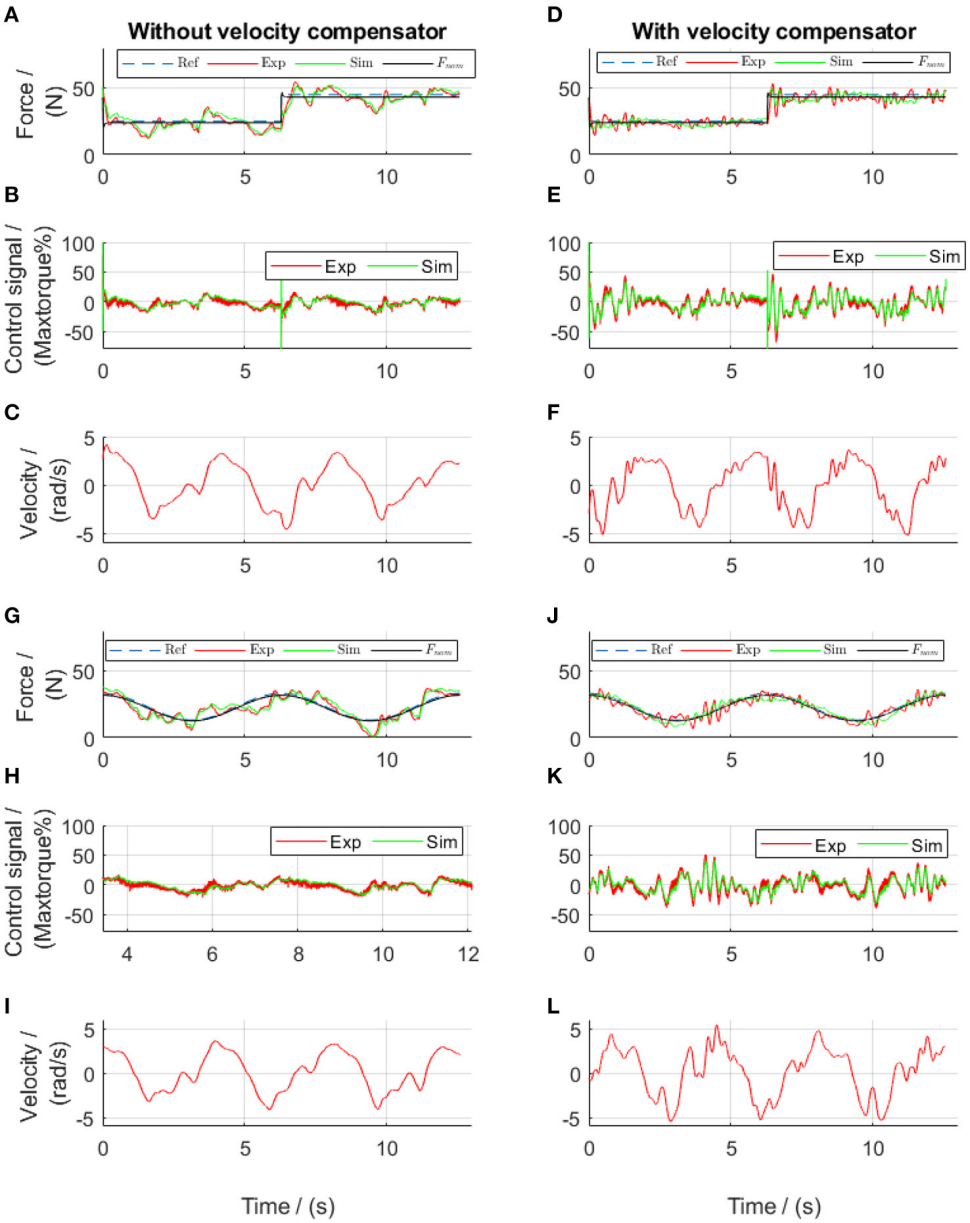
Lead algorithms regulated force control of the front thigh actuator in the dynamic tests. Subplots **(A–F)** show the results from square-wave reference test, while subplots **(G–L)** show the results from sinusoidal reference test. Ref, references; Exp, experimental values; Sim, simulation results; *F*_nom_, the simulated target.

**Figure 12:**
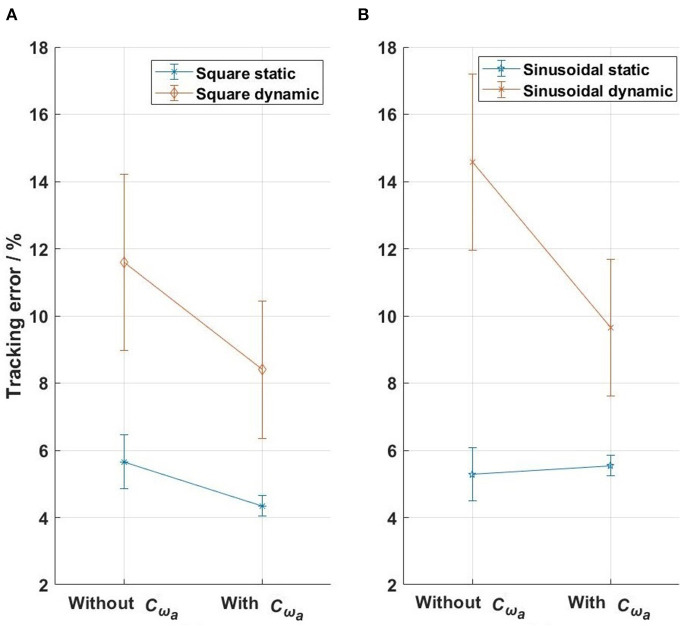
The tracking errors of the four actuators in the evaluation tests. **(A)** Square-wave reference test, **(B)** sinusoidal reference test. The bars with points in the middle show the standard deviations and the means of the errors from the four actuators.

**Figure 13:**
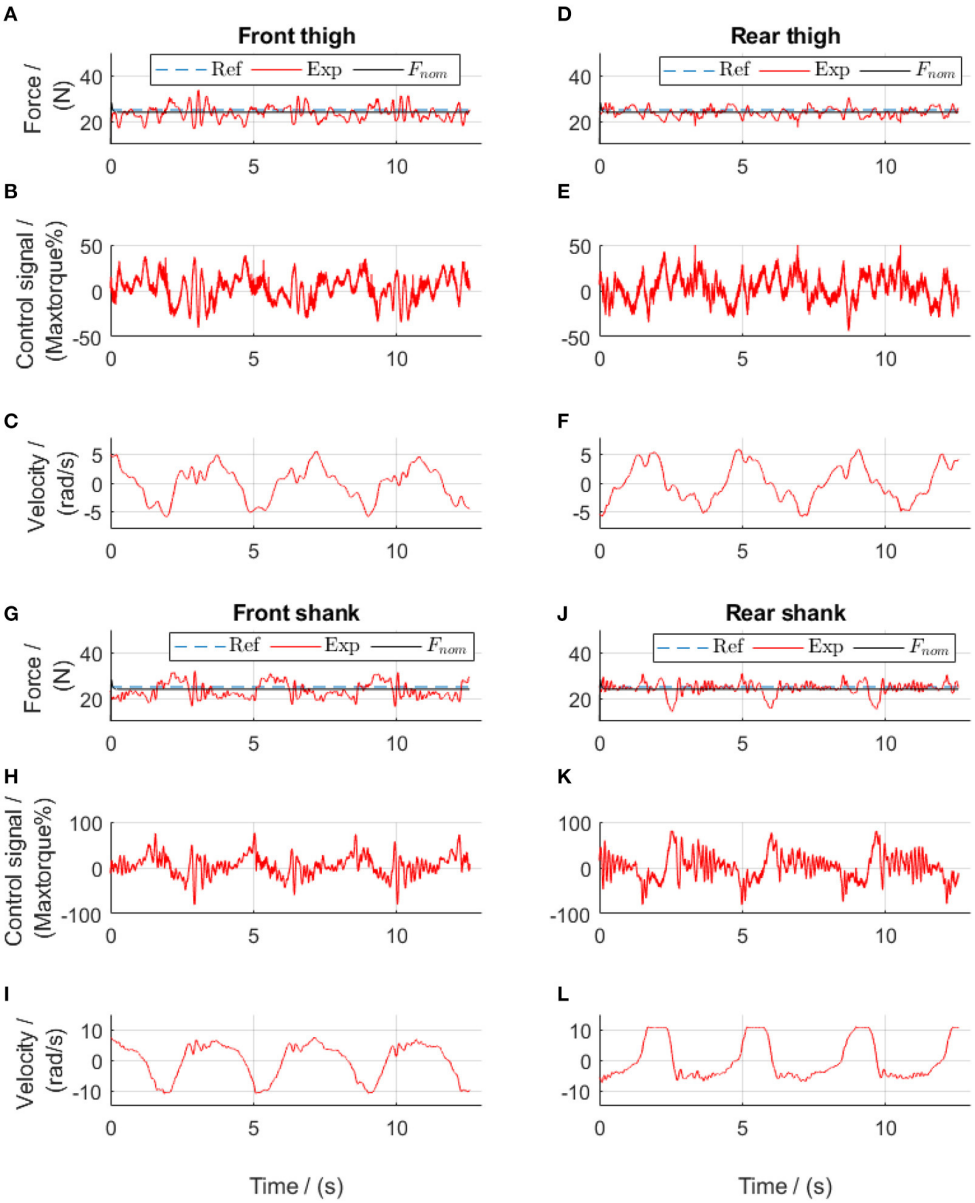
Constant-reference force control during walking using force-feedback lead controllers and velocity-feedforward lead compensators. Subplots **(A–C)** show results from the front thigh actuator, **(D–F)** from the rear thigh actuator, **(G–I)** from the front shank actuator while **(J–L)** from the rear shank actuator. Ref, references; Exp, experimental values; *F*_nom_, the simulated target. The positive angular velocity of the front actuators in **(C,L)** means that the right leg moved backwards.

The results from the parameter determination tests, followed by the controller design and evaluation tests are presented. During the tests in the active cable-driven robotic system, the four actuators were controlled simultaneously. To reduce the figure numbers, Figures 5–11 only show the representative results of the front thigh actuator, while the results of all actuators are presented in Figures 12, 13. During evaluation of the force control algorithms (section Evaluation of the Force Control Algorithms), the simulated force output *F*_sim_ and the control signal *T*_*mp*sim_ are presented in figures using green lines, serving as a comparison for the experimental results, which are presented as red lines. The force output from the nominal plant, *F*_nom_, is presented in black lines, serving as the target during the calculation of RMSE_F*exp*_. The positive angular velocity of the actuator in Figures 5C,F,I,L, 10C,F,I,L, 11C,F,I,L means the right leg moved backwards.

### Parameter Determination Tests

By trial and error, the parameters of the tentative PID controllers for both thigh actuators were tuned to be: *k*_*p*_ = 0.4, *k*_*i*_ = 0.2, *k*_*d*_ = 0.04, and τ_*d*_ = 0.1. Those for both shank actuators were: *k*_*p*_ = 0.6, *k*_*i*_ = 0.3, *k*_*d*_ = 0.06, and τ_*d*_ = 0.1. These PID controllers allowed implementation of a series of tests, which produced experimental data for determination of the system parameters. After repetitive comparisons between the simulated force *F*_sim_ and the experimental force *F*_exp_, the system parameters were determined (Table 1). The PID-regulated results when the participant walked at 0.1 m/s are presented in Figure 5. It was observed that the model yielded more similar results in the dynamic tests than in the static tests. This might be due to static friction, which was neglected in the Simulink model. Nevertheless, the simulated force outputs (green lines) present similar curves with comparable amplitudes to those of the experimental results (red lines). The NRMSE between *F*_sim_ and *F*_exp_ during the dynamic tests with the sinusoidal reference in the front thigh actuator was 75 % (Figure 5J and Table 2). The four actuators showed a mean NRMSE ≥ 70 % (Table 2), which demonstrated that the determined system parameters in Table 1 were satisfactory.

**Table 1:** System parameters.

**Geometry-determined parameters**				**Test-determined parameters**			
	**Thigh actuators**	**Shank actuators**	**Units**		**Thigh actuators**	**Shank actuators**	**Units**
*τ_*a*_*	0.0013	0.0012	s	*B_*a*_*	0.0455	0.03	Nms/rad
*k_*a*_*	−0.4	−0.2	Nm	*k_*s*_*	3000	3000	N/m
*J_*a*_*	0.0156	0.0132	kgm^2^	*k_*fr*_*	23	15	
r	0.023	0.023	m				

**Table 2:** NRMSE [goodness of fit, %, calculated using Equation (10)] between the simulated force *F*_sim_ and the experimental force *F*_exp_ during the dynamic tests.

	**Front thigh actuator**	**Rear thigh actuator**	**Front shank actuator**	**Rear shank actuator**	**Mean ± STD**
Square	68	66	72	76	71 ± 4.4
Sine	75	68	74	62	70 ± 6.0

From the point of view of force control, the tentative PID controllers produced good force tracking during the static tests (Figures 5A,G). The experimental force showed a tracking error of 9.3% in Figure 5A and 15.7% in Figure 5G. This error was considered to come from the static friction. It should be noted that in the sinusoidal force control (Figure 5G), the force output showed a phase delay of 70.6° compared to the reference. Compared to the static tests, the PID controllers in the dynamic tests failed to produce satisfactory force tracking, in that the force output was either larger or smaller than the reference (an error of 24.0% in Figure 5D and 19.9% in Figure 5J). Comparison between Figures 5D,F and also comparison between Figures 5J,L both revealed that the force output was influenced by the walking velocity, in that the force curve took a similar shape of the velocity curve.

The actuator-driven cables were observed to be loose briefly at toe off and heel strike. These problems were more obvious when the walking speed increased. The participant described it as a difficult walking because of the continuously varying force on the leg.

### Frequency-Domain Analysis for the Controller Development

Using the determined system parameters (Table 1), the transfer functions of the force output from the actuator torque command, *G*_*FT*_*mp*__ in Equation (7), and from the actuator velocity, $G_{F\omega_{a}}^{A}$in Equation (8), were obtained. The Bode diagram of *G*_*FT*_*mp*__ shows a phase shift of 150° at frequencies between 10 and 20 rad/s (black solid lines in Figure 6). The Bode diagram of $G_{F\omega_{a}}^{A}$ shows a large influence (amplitude of 54 dB) of the velocity on the force output at a frequency of 10 rad/s (red solid lines in Figure 6).

Simulation of the force-feedback lead compensator *C*_*F*_ with different parameters showed that a low *f*_*F*_ brought an overshoot to the closed-loop system *G*_*FF*_*ref*__, and a high *f*_*F*_ reduced the bandwidth of *G*_*FF*_*ref*__. The parameter *a*_*F*_ influenced *G*_*FF*_*ref*__ differently, in that the bigger *a*_*F*_ was, the lower the bandwidth was, and the higher the overshoot was. The closed-loop system *G*_*FT*_*mp*__ required a high bandwidth to secure a fast response. However, a high bandwidth requires a high sample rate in controlling the physical system. After many simulations, suitable values for the force-feedback lead controller for the front thigh actuator were determined as: *a*_*F*_ = 0.05, *f*_*F*_ = 100 rad/s, and *k*_*F*_ = −1.3. The bandwidth of *G*_*FF*_*ref*__ (black dashed lines in Figure 6) was 172.4 rad/s. The same values of *a*_*F*_ and *f*_*F*_ were used for the rest actuators, but *k*_*F*_ for the rear thigh, front shank and rear shank actuators were −2.5, −2.5, and −3.5, respectively.

With the lead controller for the force-feedback control, the influence of the velocity on the force output was reduced (red dashed lines in Figure 6), but was not yet negligible. The design target of the velocity-feedforward compensator was that its final influence on the force output, $G_{F\omega_{a}}^{C}$, should compensate as much as possible the influence of the velocity on the force output, $G_{F\omega_{a}}^{B}$. By trial and error, the parameters of the velocity-feedforward lead compensator for the front thigh actuator were determined as *a*_ω_*a*__ = 0.02, *f*_ω_*a*__ = 10 rad/s, and *k*_ω_*a*__ = −1.4. With this compensator, the magnitude of $G_{F\omega_{a}}^{C}$ is close to $G_{F\omega_{a}}^{B}$, especially at frequencies between 1 and 40 rad/s (Figure 7). The Fourier transform of the experimental angular velocity during walking at 0.2 m/s showed a maximal frequency of 20 rad/s. Therefore, the current velocity-feedforward lead compensator should be feasible for the front thigh actuator. With the combined force algorithms, the velocity (dashed lines) compared to the reference (solid lines) force had a large influence on the control signal in the overall system (Figure 8).

Using similar methods to those described above, the parameters of the velocity-feedforward lead compensators for the rear thigh actuator were determined as: *a*_ω_*a*__ = 0.02, *f*_ω_*a*__ = 10 rad/s, and *k*_ω_*a*__ = −1.2. The values of *a*_ω_*a*__ = 0.01, *f*_ω_*a*__ = 30 rad/s were used for both shank actuators, but *k*_ω_*a*__ for the front and rear shank actuators were, respectively, −2.2 and −1.8.

Frequency analysis of the loop gain *G*_*Lo*_ of the front thigh actuator yielded a gain margin of 27.4 dB (at 824 rad/s) and a phase margin of 61.5° (at 103 rad/s). The Nyquist diagram also shows the modulus margin, which was computed to be 0.79 (Figure 9). Therefore, the force control system is stable with adequate stability margins.

### Evaluation of the Force Control Algorithms

Different force references were implemented and tests performed using the force control algorithms developed in section Frequency-Domain Analysis for the Controller Development. During the static tests (Figure 10), the experimental force showed an error of 6 and 4.79% during the tests with the square-wave and sinusoidal references, respectively. It should be noted that the phase delay in the sinusoidal force control, which was 70.6° in the PID-regulated test (Figure 5G) was reduced to be 28.0° (Figure 10G) using the force-feedback lead controller. Although the participant stood still, the actuator still moved slightly. The mean velocity during the rising edge of the square pulse was 1.5 rad/s in Figures 10C,F, and the velocity during sinusoidal test was 0.01 rad/s in Figures 10I,L. Using the velocity-feedforward lead compensation, the force-tracking error was reduced by 0.6% (comparing Figures 10A,D) and by 0.01% (comparing Figures 10G,J) during the tests with the square-wave and sinusoidal references, respectively.

During the dynamic evaluation tests, the force-feedback lead controllers alone yielded improved force tracking compared to the tentative PID controllers, with a mean tracking error of 12.5% in square-wave and 15.5% in sinusoidal references (Figures 11A,G). However, similar to the force output using the PID controllers, the force output using only the force-feedback lead controllers still showed the influence of the walking movement (comparisons between Figures 11A,C, and also between Figures 11G,I). After further implementation of the velocity-feedforward lead compensators, the mean force control error reduced to 8.1% in square-wave and 9.2% in sinusoidal references (Figures 11D,J) during the dynamic tests. These results demonstrated that the control algorithms were satisfactory. The improved force tracking was obtained by sending a more dynamic control signal to the system. This dynamic control signal thereby brought a more dynamic actuator velocity. Nevertheless, during the dynamic tests, the participant reported much more comfortable walking when the velocity-feedforward lead compensator was further implemented compared to the tests without.

Similar results were obtained in all four actuators (Figure 12). The mean force-tracking errors of all four actuator-driven cables in the static tests were 5.7 and 5.3% for the square-wave and sinusoidal references, respectively, which showed that the force-feedback lead controllers produced satisfactory force tracking (mean error of 5.5%) in the static tests. But in the dynamic tests, the mean tracking errors were 11.6 and 14.8% for the square-wave and sinusoidal references (mean error of 13.2%), respectively. Further implementation of the velocity-feedforward compensators reduced the force control error in the dynamic tests to 8.4 and 9.6% for the square-wave and sinusoidal references, respectively. This showed that the force-feedback controllers with velocity-feedforward compensators produced satisfactory force tracking (mean error of 9.0%) in the dynamic tests.

In the final walking test with a constant reference in each cable (see Supplementary Material “Walk with the active cable-driven robotic system. mp4”), both thigh actuators yielded good force tracking (Figure 13). The shank actuators had velocities almost twice those of the thigh actuators, thereby having a larger influence of velocity on the force output. The slightly noisy control signals came mainly from the velocity input (Figure 8). Nevertheless, all actuators yielded satisfactory force tracking, with tracking errors from the actuators for the front thigh, rear thigh, front shank and rear shank of 12.1, 8.2, 12.4, and 8.5%, respectively, resulting in a mean error of 10.3%. It should be noted that the reference force was only 25 N, therefore the absolute mean force tracking error was 2.6 N.

## Discussion

The aim of this work was to develop an active cable-driven robotic system, and to evaluate force control strategies for walking rehabilitation using frequency-domain analysis. The experiment-assisted simulation method determined the system parameters, which provided the basis for the development of the force control strategies. The control algorithms were firstly designed using frequency-domain analysis, then simulated in Simulink dynamic models, and finally evaluated in the active cable-driven robotic system. The force-feedback lead controllers provided good force tracking in the static tests, but were not satisfactory in the dynamic tests. Using velocity-feedforward lead compensators in addition to the force-feedback lead controllers, the active cable-driven robotic system produced satisfactory performance in tracking various force profiles during walking. The novelties of the current work include using the experimental-assisted simulation method to determine the system parameters, and deducing, simulating, and evaluating lead control algorithms for the force regulation and motion-related disturbance compensation in the cable-driven system. To the authors' knowledge (based on the extensive literature review), this control strategy hasn't been published in the literature. After being validated in four cable-driven actuators in the current work, this control strategy can be applied generally in active force-controlled cable-driven robotic systems. The robotic system developed in this work has the potential to produce user-defined assistance or resistance in rehabilitation and fitness training.

The experiment-assisted simulation method determined the system parameters, which helped to understand the system dynamics, thereby developing the proper control algorithms. The dynamics of the cable-driven robotic system is complex, with the transfer function from the actuator control signal to the force output (Equation 7) as a fourth-order system. Determination of the system parameters was the prerequisite for understanding the system dynamics. Study (Jung and Bae, 2016) used system identification and linear quadratic methods to determine the plant transfer functions and the controller. Other studies (Zou et al., 2018a, 2019; Wang et al., 2020) listed the system parameters without describing how they were obtained. Using the tentative PID controllers, the current work performed several tests on the active cable-driven robotic system, where the actual force outputs were recorded. Then repetitive model simulations found suitable system parameters, which yielded similar force outputs to the experiments (the mean NRMSE ≥ 70%). This experiment-assisted simulation method searched for suitable parameters for the active cable-driven robotic system during walking, which provided the basis for the following development of the control strategies. However, there might be other suitable system parameters, and a systematic determination of the optimal parameters would require further investigation.

Based on the system dynamics, force-feedback lead controllers were developed, which yielded satisfactory static force tracking. Traditional PD controllers are often reported in the literature (Marchal-Crespo et al., 2012; Alamdari and Krovi, 2015b). The current study implemented PID controllers during the system parameter determination phase. But the phase delay observed in the sinusoidal-reference force tests motivated the development of force-feedback lead controllers. Comparing the lead controllers with the PID controllers in the current study revealed that the PID controllers produced better tracking of the square wave force, due to the inclusion of the integral term (comparing Figure 5A with Figure 10A), while the lead controllers reduced the phase delay during sinusoidal force tracking (comparing Figure 10G with Figure 5G). It is believed that by tuning the parameters of both types of force-feedback controllers, the static force tracking could be improved. The force-feedback lead controllers showed a steady-state error of 2.2%. An integrator could be further included, as demonstrated in studies (Zou et al., 2018a, 2019), to remove this error.

Frequency-domain analysis guided the development of the velocity-feedforward lead compensators, which improved the force-tracking accuracy during walking. Dynamic force control of cable-driven systems is challenging because the velocity-related disturbance cannot be removed, but can only be partially compensated (Zou et al., 2018a). Based on the frequency-domain analysis of the angular velocity of the actuator and the force output (Equations 15 and 20), the parameters of the lead compensators were straightforwardly determined by simulation. Dynamic tests on the active cable-driven robotic system showed that the disturbance was similarly suppressed as demonstrated in the simulation. The velocity-feedforward compensators in the current study were simpler than the compensator developed using the structure invariance principle (Zou et al., 2018a,b). The current velocity-feedforward compensators could be further improved by using double lead elements. Nevertheless, the mean error in all actuators was 10.3% in the force control with a constant-reference within the four cables, which can be considered negligible. The control algorithms have the potential to produce user-defined assistance or resistance in rehabilitation and fitness training (Marchal-Crespo et al., 2012; Von Zitzewitz et al., 2013) by modifying the force references of each actuator.

The limitations of this study included the mechanical and control development of the active cable-driven robotic system. The cables did not always follow the threads on the drum during the dynamic walking tests. A deflection unit as presented in Marchal-Crespo et al. (2012) is required to secure regular winding of each cable on each drum. In order to achieve accurate measurement of the pulling force on the leg, the force sensors were fixed close to the leg. However, this resulted in a situation where the force sensors moved with the leg during the dynamic tests. This movement, especially the shock at heel strike, was believed to influence the force measurements. Due to mechanical constraints, the current cable-driven robotic system could produce a maximal speed of 0.6 m/s. Therefore, the force control at high speed conditions was not investigated. Four actuator-drum assemblies with a larger drum size and proper fixation of the force sensors will be developed for accurate control of high-speed walking training. In order to compensate potentially large motion-dependent disturbances at high speed, double lead compensators could be implemented, especially for the shank actuators. The force control algorithms, although tested to be feasible in the current study, were not optimal. The force-feedback lead controllers could include an integrator to remove the steady-state errors. New control algorithms such as PID controllers with adaptive parameters, and disturbance observers as used in Jung and Bae (2016) and Wang et al. (2019) will be investigated. Many different control strategies exist in the literature for control of cable-driven systems. The control strategies presented in Von Zitzewitz et al. (2013) are also candidates for further investigation in our system in future.

The current paper evaluated lead control algorithms for force regulation and velocity compensation, with different target force profiles (sine and square wave profiles) on each cable tested. From the view of force controller evaluation, the tests were successful with straightforward results. The non-parallel cables during walking did not cause problems for the force regulation. But more work is required from the view of promotion of walking rehabilitation. Kinematic and kinetic analysis will be performed to define a suitable target force profile for each cable that jointly produce the required kinetics on the leg during normal gait (Fang et al., 2014), as a strategy to effectively guide patients in walking relearning. As another strategy to promote rehabilitation, impedance control will be implemented so as to guide patients to generate appropriate gait and to exert the appropriate force during walking training. The current work provides the important basis for the further application of the cable-driven robotic systems in walking rehabilitation.

## Conclusions

An active cable-driven, force-controlled robotic system for walking rehabilitation was developed. Using frequency-domain analysis, force-feedback controllers, and velocity-feedforward compensators were developed. The technical evaluation showed that the combined control algorithms produced satisfactory force tracking during walking in the active cable-driven robotic system. This study demonstrated that the force control algorithms were technically feasible. In future, four actuator-drum assemblies with proper fixation of the force sensors will be developed, and the force control algorithms will be further improved by adding integrator elements, implementing double-lead velocity compensators, and tuning the control parameters. As strategies to effectively guide patients in walking relearning, kinematic and kinetic analysis will be performed, and impedance control will be further implemented. The active cable-driven, force-controlled robotic system has the potential to produce user-defined assistance or resistance in rehabilitation and fitness training.

## Data Availability Statement

The raw data supporting the conclusions of this article will be made available by the authors, without undue reservation.

## Ethics Statement

Ethical review and approval was not required for the study on human participants in accordance with the local legislation and institutional requirements. The patients/participants provided their written informed consent to participate in this study. Written informed consent was obtained from the individual(s) for the publication of any potentially identifiable images or data included in this article.

## Author Contributions

JF, LM-C, and KH developed the system concept. MH assembled the active cable-driven robotic system. JF developed the control algorithms. JF and MH implemented the control programs. JF conducted the experiments, analyzed the data, and drafted the manuscript. KH, LM-C, and MH revised it critically for important intellectual content. All authors contributed to the article and approved the submitted version.

## Conflict of Interest

The authors declare that the research was conducted in the absence of any commercial or financial relationships that could be construed as a potential conflict of interest.
